# Construction of liquid metal-based soft microfluidic sensors via soft lithography

**DOI:** 10.1186/s12951-022-01471-0

**Published:** 2022-05-28

**Authors:** Yang Zhang, Haowei Duan, Guoqiang Li, Maoyu Peng, Xing Ma, Ming Li, Sheng Yan

**Affiliations:** 1 Institute for Advanced Study, Shenzhen University, Shenzhen, 518060 China; 2 School of Engineering, Macquarie University, Sydney, NSW 2109 Australia; 3 Sauvage Laboratory for Smart Materials, Harbin Institute of Technology (Shenzhen), Shenzhen, 518055 Guangdong China

**Keywords:** Microfluidics, Biosensors, Liquid metal, Soft electronics

## Abstract

**Background:**

Liquid metal (LM) can be integrated into microfluidic channel, bringing new functionalities of microfluidics and opening a new window for soft microfluidic electronics, due to the superior advantages of the conductivity and deformability of LMs. However, patterning the LMs into microfluidic channels requires either selective surface wetting or complex fabrication process.

**Results:**

In this work, we develop a method to pattern the LMs onto the soft elastomer via soft lithographic process for fabrication of soft microfluidic sensors without the surface modification, bulky facilities, and complicated processes. The combination of the interfacial hydrogen bond and surface tension enables the LM patterns transfer to the soft elastomer. The transferred LM patterns with an ellipse-like cross-section further improve the stability under the mechanical deformation. Three proof-of-concept experiments were conducted to demonstrate the utilization of this method for development of thermochromic sensors, self-powered capacity sensors and flexible biosensor for glucose detection.

**Conclusions:**

In summary, the proposed method offers a new patterning method to obtain soft microfluidic sensors and brings new possibilities for microfluidics-related wearable devices.

**Graphical Abstract:**

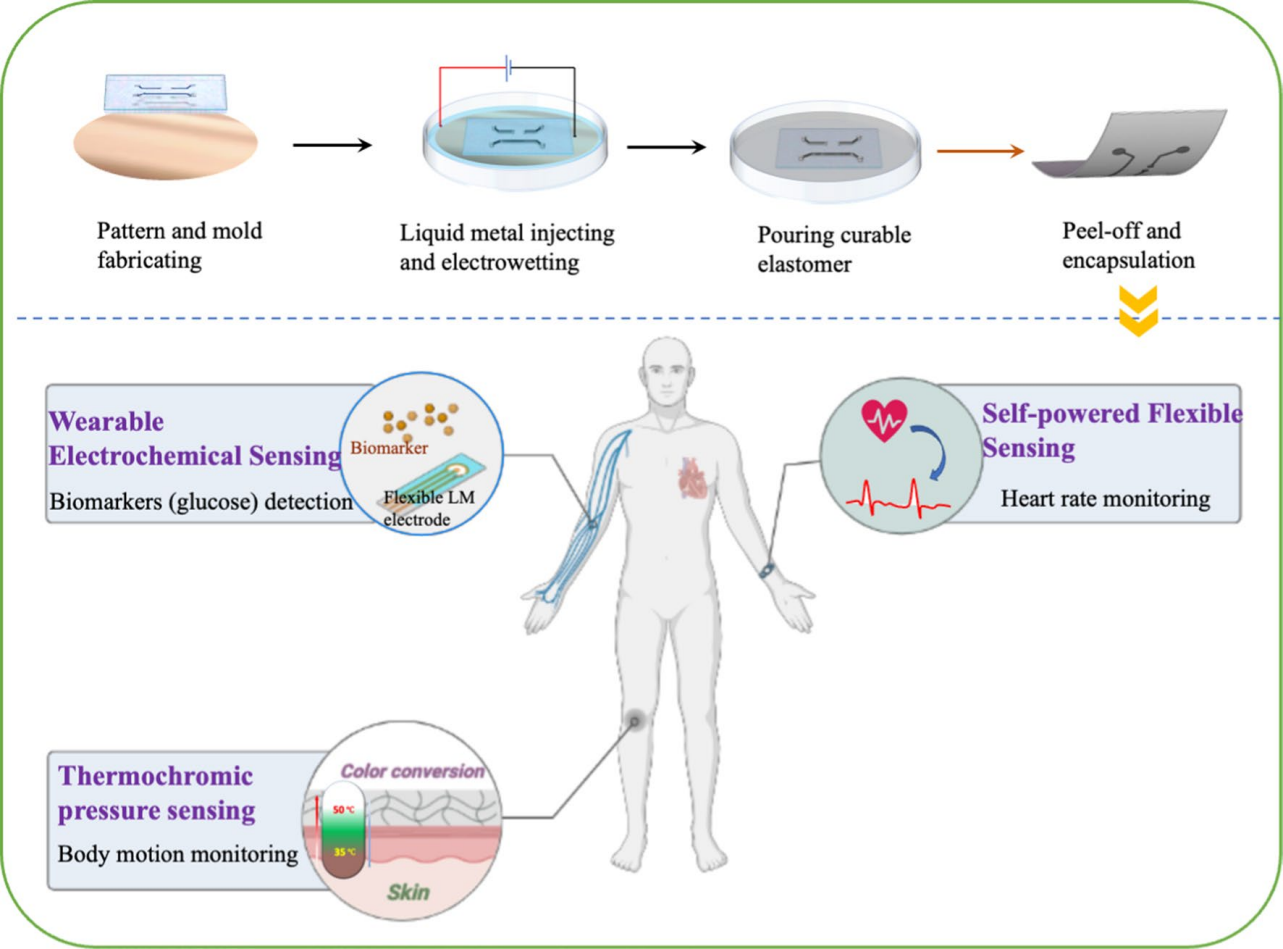

**Supplementary Information:**

The online version contains supplementary material available at 10.1186/s12951-022-01471-0.

## Introduction

Motivated by the increasing demand of lab-on-a-chip applications [1] and point-of-care diagnosis [2], microfluidics has been received great interests in the past decades due to superior advantages of low energy and reagent consumption, compact size, portability, and rapid detection [3]. Soft microfluidics enables conformal contact with human body and has been integrated with electronic circuits for electrochemical analysis of sweat [4] and blood flow mapping [5]. Soft microfluidics with rigid metal circuits cannot suffer from the larger strain as the large deformation causes the cracks on the rigid metal circuits, resulting in the loss of electrical conductivity.

Instead, liquid-phase conductors are compatible with microfluidics for all-soft, elastically deformable, and even self-healing electronics [6, 7]. Among the liquid-phase conductors [8, 9], gallium-based liquid metal (LM), such as EGaIn (75.5% gallium and 24.5% indium by weight) and Galinstan (68.5% gallium, 21.5% indium, and 10.0% tin by weight), stands out as an excellent candidate in virtue of high electrical conductivity and deformability [10]. In addition, a thin (~ 1–3 nm thick) oxide layer of LMs shows high affinity and adhesion to soft elastomers, improving the electrical continuity during significant deformations [11]. In the presence of LM circuits, the soft microfluidic electronics can maintain its conductivity under large deformations. To date, LM can be integrated into the microfluidics [12] and LM-based soft microfluidics has been also used for wearable electronics, biomedical sensors, and transient circuits [13, 14].

A variety of methods are available to print LM on soft elastomers, which have been well reviewed elsewhere [15]. One common method is the direct-write printing of LM, which offers the superior ability to quickly pattern liquid metals on demand [16–20]. However, due to the high surface tension and the rheological behavior of LM, it is difficult to extrude LM into filaments. Thus, printing LM requires very close contact between surfaces and LMs to induce the shear or tensile forces for filament generation [21]. In particular, the properties of LM can be regulated by adding different nanoparticles [22, 23] like iron (Fe) and nickel (Ni) to regulate the adhesion and wettability of liquid metal. Suspending LM particles into a polymer matrix is another solution to modify the rheological behavior of LMs [20]. However, to form a conductive path, post-processing such as mechanical sintering or laser sintering is required to “sinter” the LM particles. The sintering processes can be used to break the oxide layer of the LM and overcome the high contact resistance between LM particles. Unfortunately, it also increases the potential possibilities of damaging the structures of soft substrates and LM patterns.

Apart from LM modification, substrate modification is another important approach to develop LM flexible electronics. The substrate can be modified for LM selective wetting by patterning a substrate with very thin layers of solid metals (such as copper [24] and gold [25]) such that the LM can amalgamate the surface of solid metals. Despite its high resolution, patterning solid metal requires time-consuming and labor-intensive processes including photolithography and metal sputtering. Additionally, chemical modifications are required for selective surface treatment to form hydrophilic surfaces, so that LM can be better adhered to the designed areas [26–28]. Besides, the process of photolithography is the most common process to fabricate LM circuits currently. However, the large-scale promotion of LM soft electronics fabricating still needs to explore because of the mentioned high-cost and inefficient processes [29].

Our previous work for fabricating microfluidics channels using liquid metal-based amalgamation-assisted lithography [30], a new phenomenon was found that when the channel size is reduced to around 1 mm, the LM can be detached from the liquid metal stamp to form the conductive pathway on the elastomer surface. We discovered the mechanism behind this new discovery and applied this method for the fabrication of microfluidic sensors in this study. Furthermore, we proposed a soft-lithographic technique based on electrochemically prepared LM stamps for the fabrication of soft microfluidic electronics by integrating microchannel fabrication and LM filling into one step. The curable polymers can duplicate the patterns on the LM stamps and detach the LMs from LM stamps due to the interfacial surface tension and hydrogen bonds. Similar to wooden stamps, the LM stamps can also be refilled by LM “ink” for repeatable and large-scale use. This method is theoretically applicable to any curable elastomers for the generation of soft microfluidic electronics. Furthermore, a series of functional tests were conducted to evaluate the stability of the soft microfluidic circuits when experiencing different kinds and extents of deformation. Based on the excellent performance of the as-prepared LM microfluidic circuits, we further demonstrated the development of three sensors: a thermochromic stress–strain visualization sensor, a self-powered sensor and a flexible biosensor for glucose detecting. This technique shows great potential as a new fabrication method in the field of soft microfluidic electronics.

## Results and discussion

### Advanced liquid metal-based stamp for soft microfluidic circuits

**Fig. 1:**
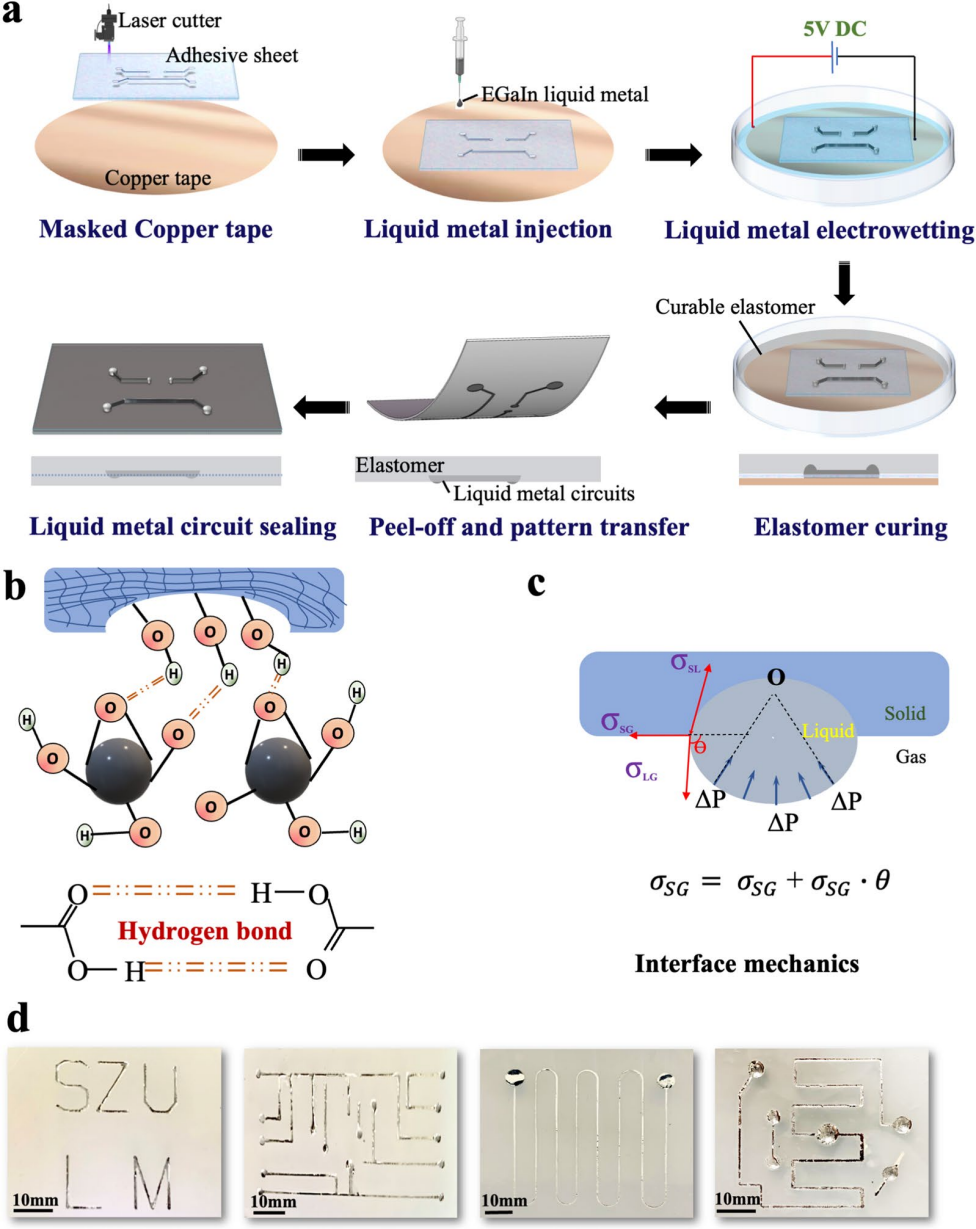
Fabrication of flexible circuits based on LM stamp. **a** Schematic of liquid metal circuit fabrication process. **b** The mechanism of demold stage including hydrogen bonding between LM and substrate and **c** interfacial tension. **d** Transferred liquid metal patterns on soft substrate (Ecoflex 00-30)

Figure 1a schematically illustrates the illustrations of the fabrication of soft microfluidic circuits based on the soft-lithographic method. LM was first patterned on the copper surface using the previously reported amalgamation-assisted lithographic technique [31, 32]. To obtain the desired LM stamp, an adhesive sheet with a thickness of 200 μm was engraved by the laser to get the designed patterns and then attached to a petri dish covered with a layer of copper tape. Afterward, LM was dropped on the masked copper surface, followed by the addition of the NaOH (1.0 mol/L) solution to remove the oxide layer (Ga_2_O_3_) on the surface of the liquid metal. In the next step, a 5 V DC voltage (copper tape was connected to the cathode, and NaOH solution was connected to the anode, respectively) was employed to allow the electrochemical-enabled reactive-wetting and spreading of the LM on the copper surface. The behind mechanism of LM spreading is that the applied electric field drives the positive and negative ions to move towards the corresponding side of the LM droplet, inducing an equal and opposite surface charge on the conducting surface. The tangential electrical stress exerted within the electric double layer leads to the imposed shear stress on the LM surface, which induces the streamline inside the LM droplet [33]. So that a local flow is generated near the contact line of the LM droplet on the Cu substrate, thus yielding a CuGa_2_ precursor film on Cu. Since interfacial energy on CuGa_2_ surface is less than that on Cu surface, the droplet exhibits similar directional locomotion (Additional file 1: Fig. S1). After fully removing the NaOH solution, the oxide layer of LM formed and then curable elastomers [34] (e.g., PDMS, Ecoflex, etc.) were poured into the LM stamp to replicate the designed patterns. During the process of curing, there are hydrogen bonds formed between the polymer chains, as well as between the elastomers’ surface and the oxide layer (Ga_2_O_3_) of LM, which provide a robust interfacial bonding. (Fig. 1b). On the other hand, owing to the tight touch between the LM and the liquid elastomer, the surface of cured elastomer will form a groove” by replicating the LM surface, as shown in Fig. 1c. This ellipse-like cross-section can help to increase the interfacial adhesion work between the liquid and solid interface from the surface tension of LM, which is credited to a smaller apparent contact angle *θ*_app_ as shown in Additional file 1: Fig. S2. After peeling off, the LM patterns were partially transferred from LM stamp to the curable elastomers. Then, in order to sealing the LM patterns for further applications, the substrates would be placed in another petri dish and we poured the same kind of curable elastomer until the LM patterns were completely covered. When it solidified into a whole, a sealed LM conducting circuit could be obtained simply.

Figure 1d shows the liquid metal circuits on the soft substrates with complex patterns produced by the liquid metal stamp. This method can produce microfluidic channel and fill LMs in one simple step without the photolithographic process and LM injection.

### Mechanism of soft-lithographic fabrication of soft circuits

**Fig. 2:**
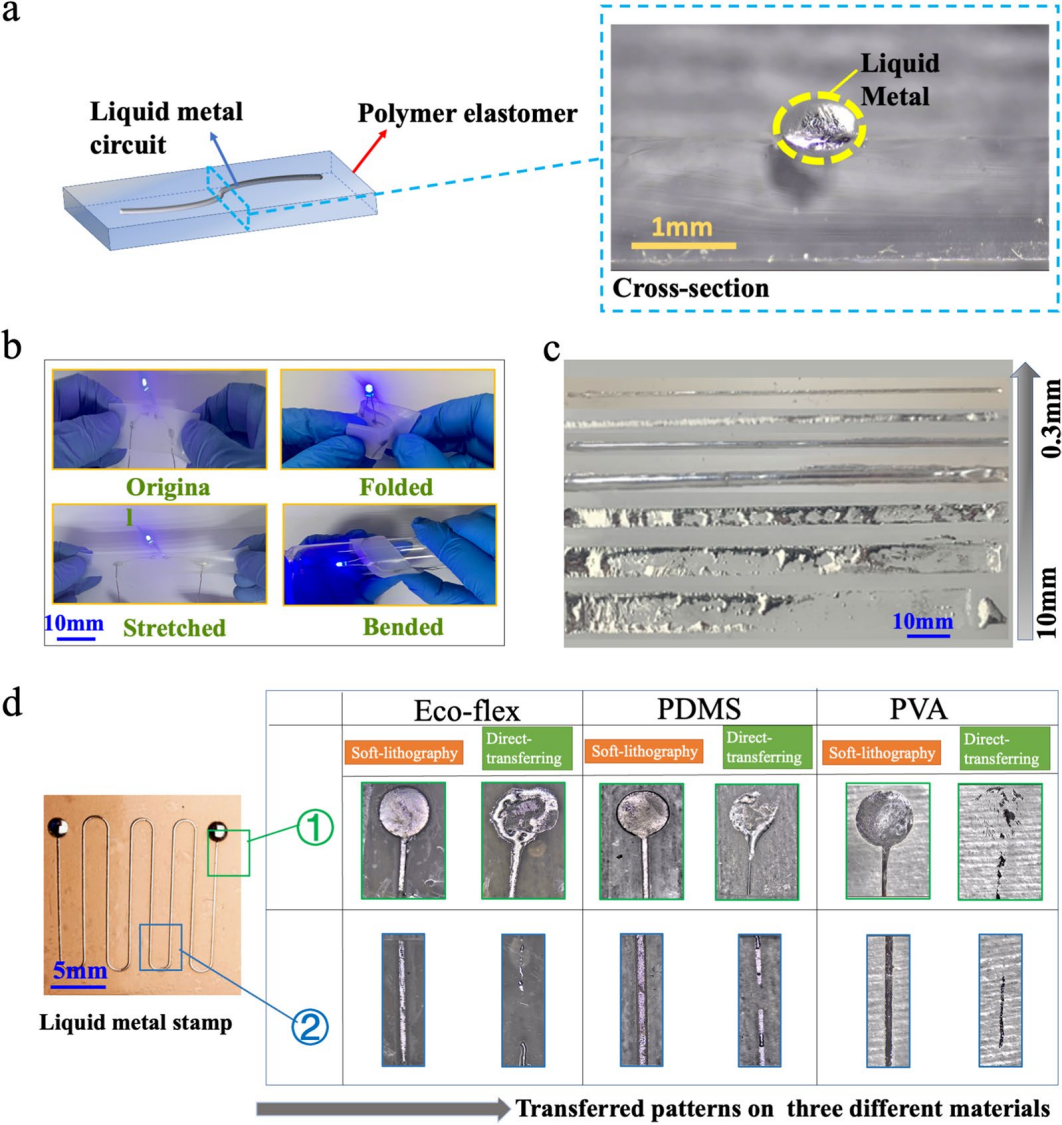
Characterizations of LM circuits. **a** The microscopic image showing the cross-section of LM circuits on the PDMS substrate. **b** Demonstration experiments on the stability of printed LM soft circuits when experienced different deformation forms: folded, stretched, and bended. **c** The images of the LM stamp and fabricated LM circuits with the different widths. **d** Characterization of LM circuits by two methods on three different substrates (Ecoflex, PDMS and PVA-H)

To investigate the underlying mechanisms of fabricating the LM microfluidic circuits by this method, its cross-section shape, size features, and universality was studied by an optical microscope. Figure 2a provides the optical microscope image of the cross-section of a LM circuit (linewidth = 0.5 mm), which shows that the LM lines from the method have a three-dimensional structure in space. The ellipse-like cross-section has a great advantage in keeping LM circuits connected when experiencing large-scale deformation because LM can flow in a larger size space as shown in Fig. 2a.

The size limitation of the circuits is one of the most important factors related to soft electronics. To investigate the feature size limitation of the fabricating method, we fabricated a group of samples with different widths of LM lines, and these LM lines with a decrease in linewidth (*d*) from 10 to 0.3 mm were duplicated on the Ecoflex substrate (Fig. **2****c**). Obviously, LM lines on the Ecoflex substrate show better integrity with the decrease of linewidth *d* (mm). The LM lines on the Ecoflex substrate reveal fracture and overflow when the *d* is greater than 2 mm. With *d* decrease to 2 mm, the structure of the LM line on the Ecoflex can maintain excellent integrity and continuity, which is vital to the further step on fabricating soft electronics. To analyze the connection between the line width and paste effect, we assume that the surface shape of LM circuits is a circular arc due to surface tension on liquids, as shown in Fig. 2a. According to the Laplace theory, a fluid with a bent surface will be under an additional pressure (Δ*P*) due to surface tension. and the direction of the force points to the center point of curvature (o). Then, the relationship between Δ*P* and* r* was obtained as: $\Delta P\propto \frac{1}{d}$ (see Additional file 1: S1). Since Δ*P* is reversely proportional to LM linewidth, a larger Δ*P* induced by the smaller size of LM circuit is conducive to maintaining the integral structure of LM circuits. Therefore, a smaller *d* has a better ability to duplicate LM circuits from the LM stamp, which can be observed from Fig. 2c. In this work, the size limitation is 0.3 mm due to the resolution of laser engraving and high surface tension of the LM. However, the size limitation for electronics printing can be further improved with the advanced laser fabrication or other lithographic method [29].

We also investigate the effect of LM height on the patterning process. The LM height can be controlled by the volume of injecting LM. With the increase of LM volume from 10 to 40 µL, the LM height increased from 1 to 3.4 mm (Additional file 1: Fig. S3). We found the incompleteness and rupture in the LM circuit with the height of 1 mm. When the LM mold height is over 2.5 mm, the size of the copied LM circuits is larger than the designated size due to the high surface tension of LM (Additional file 1: Fig. S3). Therefore, the height of channel should be around 2 mm to obtain a higher quality of LM pattern in the case.

Compared with the direct- transferring method, this fabrication method combines the surface adhesion between the oxide layer of LM and the soft elastomer with the additional pressure induced by the a forementioned groove structure. The groove structure contributes significantly to produce an apparent contact angle *θ*_*app*_, which is smaller than the contact angle *θ* in the plane as shown in Additional file 1: Fig. S2. Generally, the adhesion ability can be used to measure the attraction between liquid and solid or required energy of separate interface reversibly [29], and the work of adhesion of LM to a solid surface *W*_*SL*_ can be calculated by the Young-Dupre equation as the following ${W}_{SL}={\sigma }_{LG}\left(1+\mathrm{cos}\theta \right)$ where *W*_*SL*_ is the work of adhesion between solid–liquid interface, σ_*LG*_ is the surface tension of the liquid–gas interface, *θ* is the contact angle. It can be concluded that larger surface tension and a smaller value of contact angle will produce a stronger work of adhesion, which lead to a better performance in the adhesion and wetting abilities of the liquid (LM) to a solid surface (substrates). Figure 2d shows the LM flexible circuits made by our soft-lithographic method and direct-transferring method. A variety of curable polymers, such as Ecoflex, PDMS, and polyvinyl alcohol hydrogel (PVA-H), were used. It can clearly illustrate that LM circuits from the soft-lithographic method have a better structural integrity, roughness, and precision demonstrating our new method can be used to fabricate liquid metal electronics on the different flexible substrates. In contrast, samples fabricated by direct-printing method appear have worse duplicating capability, for instance, the LM circuits show exhibit fracture, spill, and or escalation.

### Performance of fabricated soft microfluidic circuits upon mechanical stress

We next evaluate the electro-mechanical performance of LM-based soft microfluidic circuits. Due to the excellent stretchability of the substrate (curable elastomers) and LM, these real flexible electronic properties of the fabricated soft circuits were investigated by electrical tests. In this section, we selected circuits with linewidth *d* = 0.8 mm and length *L* = 60 mm for testing.

**Fig. 3:**
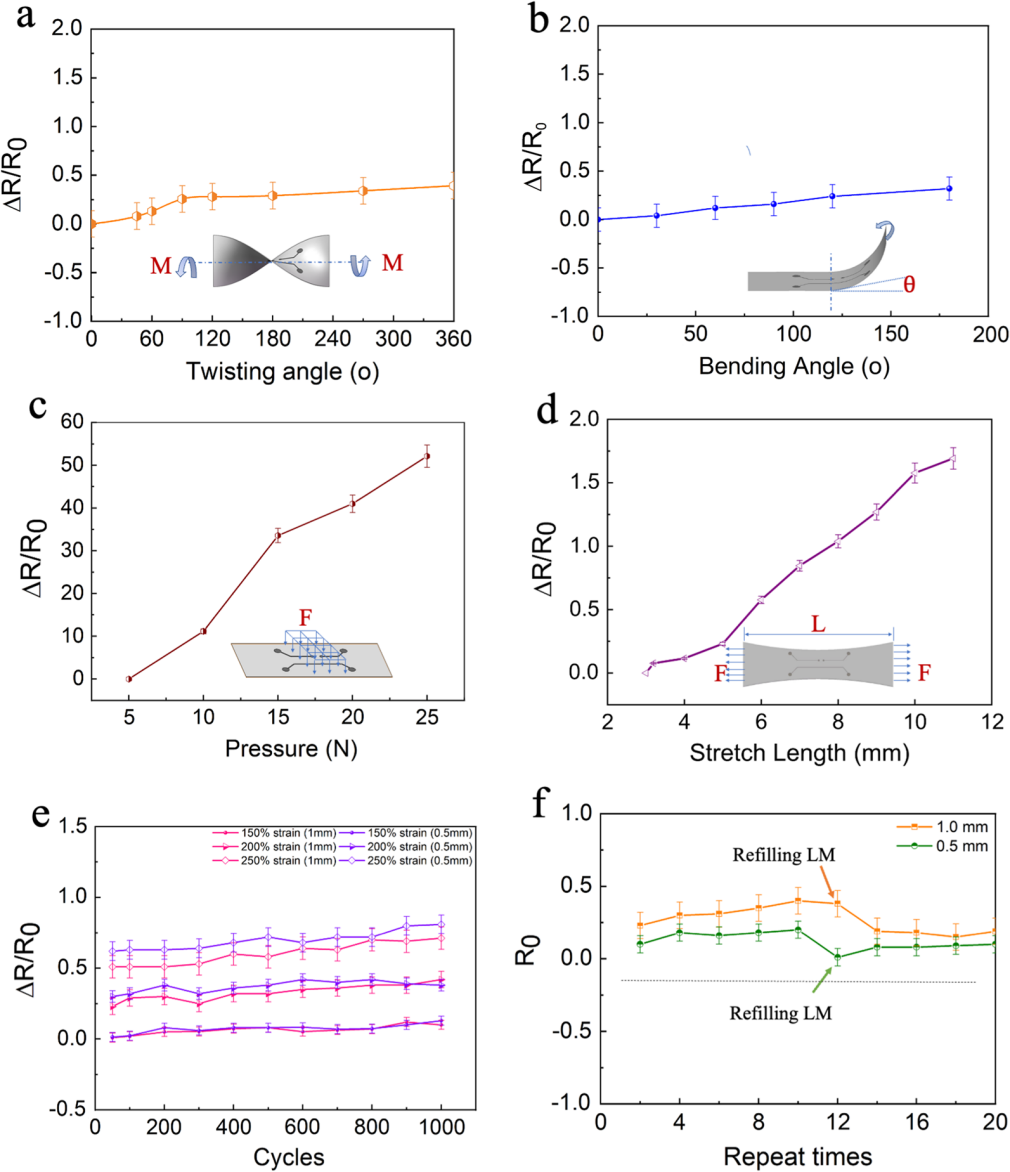
Electrical tests for the deformation response of LM circuits, showing changes in resistance of the LM line in response to different **a** twisting angles from 0° to 360°, **b** bending angles from 0° to 200°, **c** pressure from 5 to 25 N, **d** stretching length from 3 to 12 mm, **e** the cycles-RRV curve of different linewidth LM circuits under different strain levels, **f** the changes in initial resistance *R*_0_ after reusing the mold and refilling LM into the stamps

Figure 3a gives a typical twisting test for the electrical response, the LM circuit was twisted uniaxially with twisting angles from 0° to 360°. The relative resistance variation (RRV, calculated as Δ*R*/*R*_0_, where ΔR is the relative increment in resistance, *R*_0_ is the initial resistance value of the LM circuit) shows a slight increase (< 35%) even at the maximum twisting angle 360°. A similar case can be found in the following bending test, where RRV only increased by 30% (Fig. 3b). It can demonstrate that the good electrical stability of the LM circuit under twisting and bending conditions, which was attributed to the fluidity and the intrinsic stretchability of LM.

Figure 3c and d show the pressing and tensile tests. With the force on the LM circuit surface increasing from 5 to 25 N, the value of RRV experiences a significant rise by ≈ 5000%. By stretching the LM soft electronic circuit from 3 to 12 mm, the RRV of the LM circuit will increase from 0 to 170%. The RRV difference between pressing and tensile test was caused by the applied pressures in the two tests. The applied pressure (0.64–3.18 × 10^7^ Pa) in the pressing test is ~ 100 times higher than that (0.67–8.3 × 10^5^ Pa) in the tensile test (Additional file 1: S2). The higher pressure leads to a larger RRV.

To evaluate the electrical stability, the above samples were then subjected to the repeated extension deformation. Furthermore, to ensure the rationality of the results, we selected two different LM linewidth of 1 mm, 0.5 mm, respectively, and gradient strain rate at 150%, 200, 250%, separately. The testing results were illustrated in Fig. 3e (the red line represents 1 mm wide circuit, the blue line represents 0.5 mm wide circuit). It is obvious that even after 1000 stretching cycles, the change of RRV is only about 10%. That means these fabricated flexible LM circuits have great potential to apply to soft electronics because of their excellent stability. Figure 3f also shows the recyclability of the LM soft circuits. We found that the LM stamps can be reused and recycled several times. We tested two different circuits (orange line: 1.0 mm linewidth, green line: 0.5 mm linewidth) and measured the initial resistance value *R*_0_. After repeating the same process about 10 times and the value of *R*_0_ keeping slightly increasing, the *R*_0_ of both two circuits appears to remarkable decline. Because the mold still maintains original shape and structure, we can quickly refill LM without any modification and process. When we used syringe to inject LM into the designed circuits in the mold and refilled the channels, the *R*_0_ came back to the initial level again. Through the refilling tests, this fabrication method for LM soft circuits has the great potential for large-scale manufacturing of LM microfluidic circuits in a material-saving way.

### Thermochromic sensors

Based on the high thermal conductivity and electrical conductivity of LM-based microfluidic electronics [35], we explored a sensing concept of thermal-dynamic conversion. Due to the soft and stretching performance of the LM microfluidic circuits, the geometrical shape of LM will change along the deformation of the elastomers, as a result, the resistance of LM circuits changes accordingly. Theoretically, when a LM line (length *L*) generates a deformation Δ*L*, the resistance increases from its initial value *R*_0_ following:$\Delta R={R}_{0}{(\frac{\Delta L}{{L}_{0}}+1)}^{2}$. Joule heating *Q* of the LM ($\mathrm{Joule law }:\Delta Q={I}^{2}\cdot \Delta R\cdot t$, where *I* is the DC current value, *t* is the conduction time), therefore, could deliver thermal energy locally when the power supply is connected. In other words, the LM circuits were used to be a heater in response to deformation. We employed the principle to create a thermochromic sensor.

**Fig. 4:**
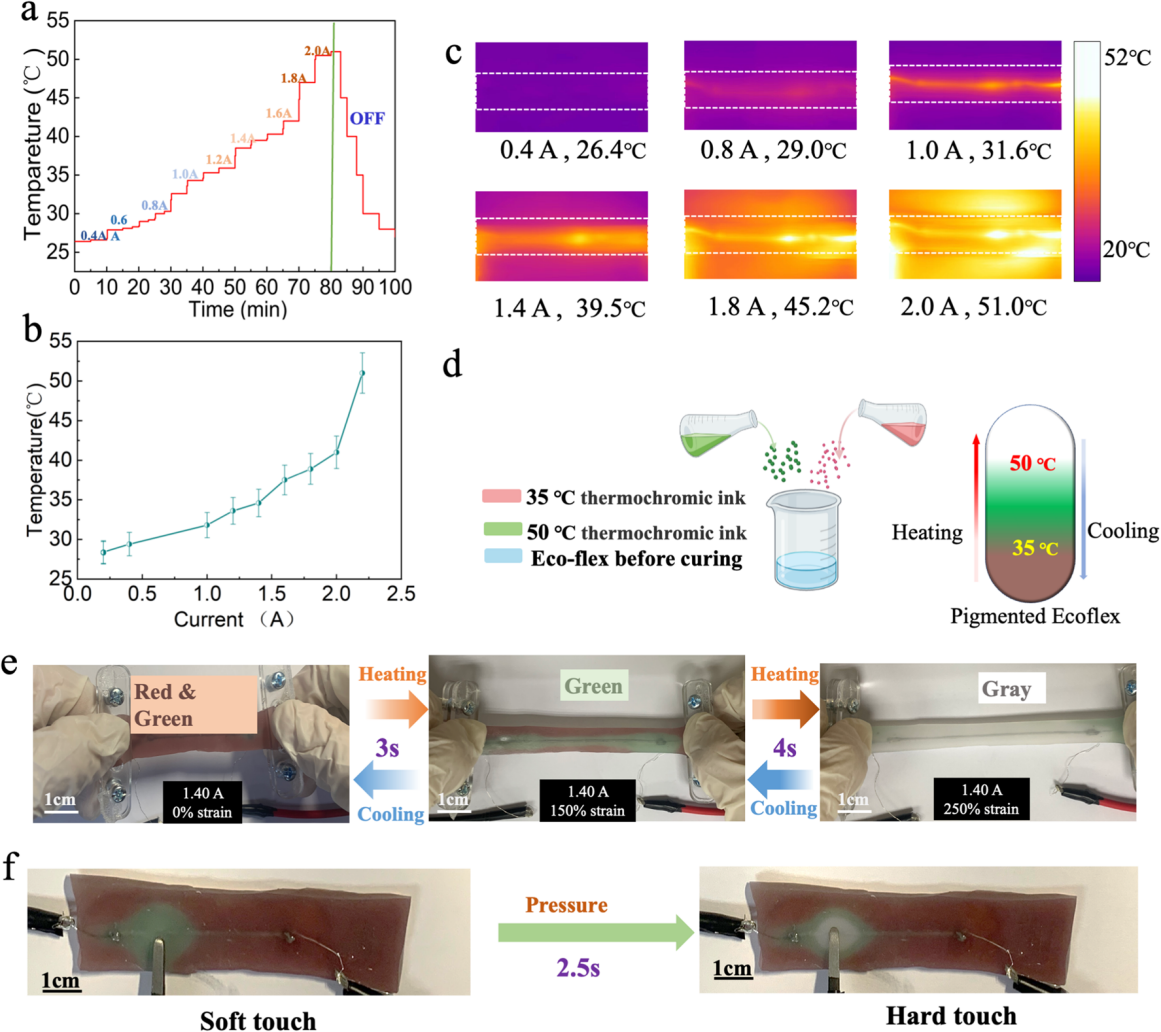
The Joule heating properties and application of LM-based thermochromic sensor. **a** The stepwise temperature changing curve with the applied DC increasing from 0.4A to 2.0 A. **b** The relationship curve between the applied current and temperature. **c** The corresponding infrared thermal images of LM circuits. The temperature values represent the average temperatures of the LM line. **d** Schematic diagram of mixing two thermochromic powders. **e** Photographs of the color-changing process with the increase of strain level from 0 to 250%. **f** Photographs of point press leads to color changing between soft touch and hard touch

Firstly, to test the heating performance, we connected a DC power supplier to a LM circuit sample (LM line width is 1 mm) and utilized an infrared thermal camera to monitor temperature changes as shown in Fig. 4a. When increasing the current from 0.4 to 2.0 A, the temperature of the LM circuits jumped from the initial room temperature of 26.4–51 °C. Once the power supplier was turned off, the temperature would experience a sharp decrease from the peak point of 51 °C down to room temperature. Figure 4b depicts the relationship between the applied current and the temperature of LM circuits, and photos obtained by the camera under different applied currents are represented in Fig. 4c.

To fabricate the thermochromic sensor, we mixed two different thermochromic powders into the Ecoflex (Fig. 4d). The first thermochromic powder is red color at room temperature but changes to translucent grey color at 35 °C. The temperature triggers the color change of another thermochromic powder is 50 °C (green turns into grey). The color map in Fig. 4d indicates the change in the color of the elastomer from red & green (T < 35 °C) to green (in the absence of red, 35 °C < T < 50 °C) and finally grey (in the absence of green and red, T > 50 °C).

Figure 4e gives a full process of color change in LM circuit when the current *I* is 1.40 A and LM line width is 1 mm (50 mm in length). With the strain level increasing from 0 to 200%, the color of the sensor changed from red and green to green within 3 s, and finally to grey because of the rise of Joule heating *Q* with the increase of electrical resistance *R* in about 4 s. At the same time, the color-change is a reversible process. Once the circuit was released, the color would change back to the original state in a short time. Figure 4f shows the results of a pressure test for the sensor. We applied different levels of external forces on one point of the sensor surface. An obvious color transformation was observed, and the color of the touchpoint changed into green (soft touch), and grey (hard touch). In addition, the response time of temperature changing when suffering pressure had also been studied, it took under around 2.5 s for the red and green color changing to green color, while the changing from green to grey spent about 1 s. Furthermore, we could pay attention to the thermochromic substrates, firstly. More different thermochromic powders can be employed to label more temperature gradients, thus the sensor can behave higher sensitive towards deformations. On the other hand, more complex and dense circuits can be designed to produce a higher resolution of color-changing response [36, 37]. To sum up, the sensors based on LMs can not only detect the stretching length, but also sensitively respond to the local pressure by naked eyes without the need for the advanced multimeters. This thermochromic sensor opens a new window for the development of visual pressure sensors to monitor the motion of human body.

### Self-powered soft sensors

**Fig. 5:**
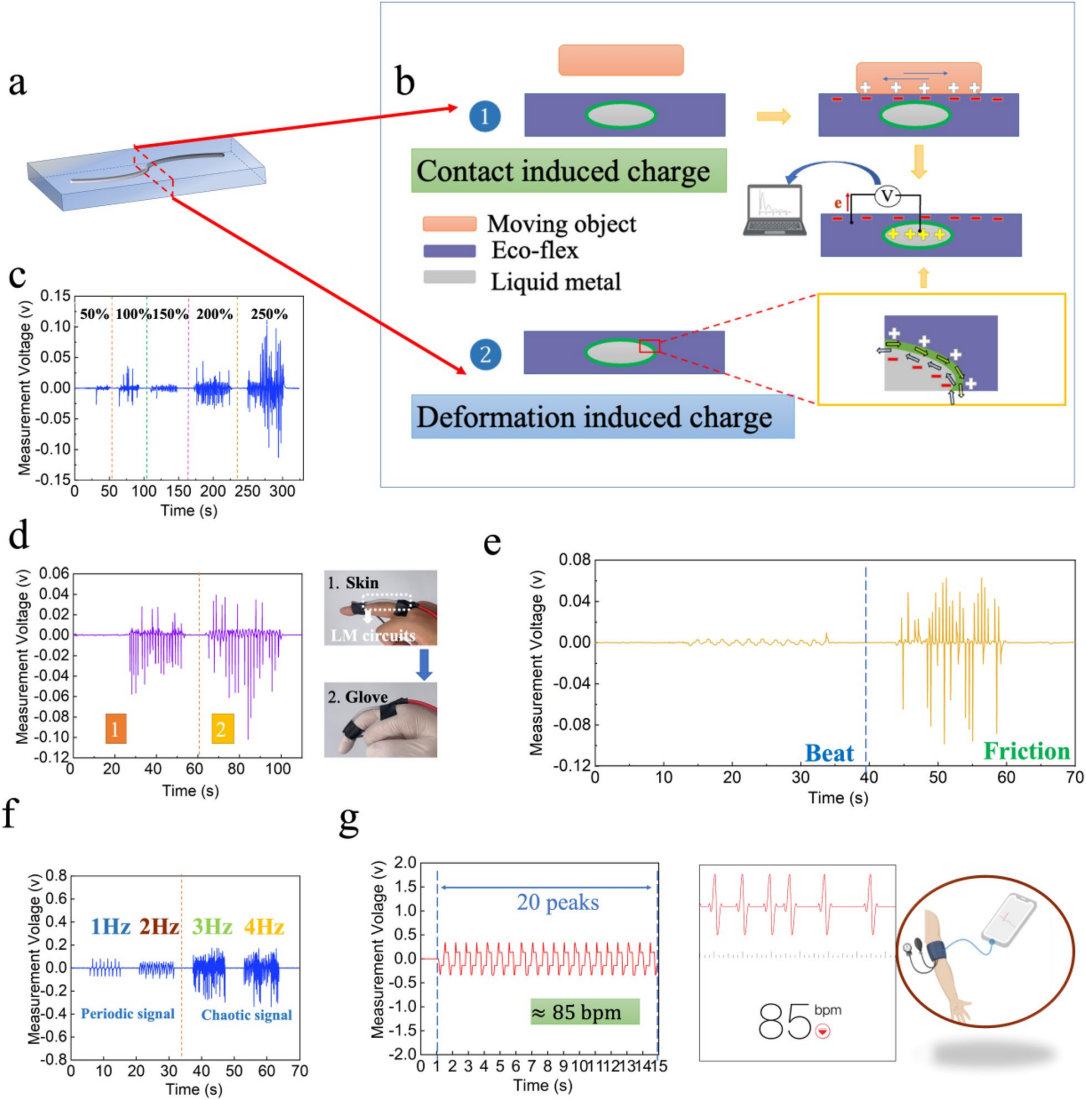
Application of LM-based flexible circuits in self-powered sensors. **a** The diagram of LM circuits with linewidth 1 mm and length 4 cm. **b** Working mechanism of charge transfer and electric potential generation. **c** The voltage signals of the LM-based sensor under the increasing strain levels. **d** The voltage signals of the LM-based sensor when adhered to human skin and rubber glove, respectively. **e** The electrical responses for different forms of motions (beat and friction). **f** Different frequencies frictions and corresponding voltage signals with two forms: periodic and chaotic waveforms. **g** Real-time monitoring data of human arterial pulse obtained by the sensor and commercial detection software, respectively

Recently, a new concept of triboelectric nanogenerators (TENGs) is developing that combines contact-electrification and electrostatic induction effects to generate electricity from various mechanical stimuli, such as friction, vibration, beat, and expanding/contracting motion [38–40]. Similarly, LM-based microfluidic sensors fabricated by our method (Fig. 5a), with high conductance at larger deformation, longer contact superficial area between LM and the electronegative surface of substrates (e.g., PDMS, Ecoflex), have innate advantages to be a soft self-powered sensor. The most used materials including human skin in our daily life tend to be positively charged when contact-electrification takes place. Therefore, we selected Ecoflex with a relatively high negative potential upon electrification as the materials of the sensor. The contact-electrification effect occurs between LM and surrounding materials once the moving objects touch the surface of the sensor, and the two surfaces will generate different charges (Fig. 5b①). To balance these static charges on the surface of substrates, there is a movement of electrons reduced between the LM surface and substrates’ surface. In addition, the relative motion between LM and substrate will also generate opposite charges on the interface of the LM-substrate (Fig. 5b②). Therefore, the movement of target objects and the deformation of the sensor can both be detected from either open-circuit voltage or the generated alternating current.

In this experiment, we acquired data by testing the open-circuit voltage between the substrate and LM. A tensile test was implemented to further investigate the response of microfluidic self-powering sensor to stretch motions. With the increase of strain rate from 50 to 250%, the relative displacement between LM and substrates increases dramatically leading to the increase of charges. The results showed that the generated voltage increased from approximately 15 mV to over 100 mV, demonstrating the strain sensing ability of LM-based microfluidic sensors without the external power supplies (Fig. 5c). We also explored the sensing ability for monitoring other real body motions as a wearable sensor. Figure 5d shows a finger bending test, in which we attached the LM-based sensor on one human finger. Bending the finger would induce the friction coupled with stretching motion between LM and skin, causing the displayed coupling voltage signals. Since the insulators has a good capability of storing charge, the sensor on the hand with a rubber glove generates a higher average voltage based on the friction between the rubber and the sensor, which suggests that the soft microfluidic sensor can distinguish the different surfaces based on their contact electrification.

Considering that sensors need to collect different forms of external excitation signals like different forms of human body movements, we thus monitored different external excitations (beat and friction) by measured voltage signals as shown in Fig. 5e. It can be observed that very different waveforms displayed under different excitations. The beat motion produced a regular waveform while the friction motion generated a higher voltage [41]. The reason for this differentiation is that beat motion provides a discontinuous, single-track, and regular excitation, which lead to a periodic relative displacement between LM and substrate. Meanwhile, friction motion gives a continuous and reciprocating excitation, in this case, a degree of electric charges accumulation occurred, which generates chaotic alternating voltage with higher value.

We further studied the more characteristics of LM-based sensor under different friction frequencies as shown in Fig. 5f. The results showed that lower frequency (1 Hz, 2 Hz) of friction produced stable and periodic signals while higher frequency (3 Hz, 4 Hz) of friction produced chaotic voltage signals with higher values of alternating voltage, which is ascribed to enhanced accumulation and flow rate of charges caused by higher and faster relative displacement between LM and substrates [42]. This interesting phenomenon could help to achieve two different functions of the sensor in various application areas, it can accurately detect low-frequency motions and be utilized as an energy-harvesting component or an electrical generator when experiences higher frequency friction. Finally, we also tried to employ the sensor to detect human arterial pulse as shown in Fig. 5g and Additional file 1: Fig. S4 and obtained a group of clear and accurate pulse period voltage signals. When we compared the signals with resting heart rate data obtained from a commercial software, it can be found that data gathered by the two different methods that monitor heartbeats indicate a similar outcome.

### Flexible electrochemical biosensor for glucose detection

The detection technique for glucose, one of the most common biomarkers, is important and meaningful in the fields of disease diagnosis, health monitoring, food and so on. Currently, most of glucose detecting methods require many time consuming steps, regents and samples [43]. Wearable and flexible biosensors have been developed since they allow for real-time and point-of-care detection of the individual’s physiological signals in a simple and comfortable manner [44, 45]. Also, this kind of biosensors based on electrochemical technique offers a number of advantages over other methods such as better accuracy and reliability. However, there are also some challenges while design and fabrication of wearable electrochemical biosensors including mechanical flexibility for electrodes. On the basis of its excellences in electrical conductivity, physical stability and flexibility, processability, the LM-based electrodes are expected to be an attractive candidature for biosensing applications to address challenges from commonly adopted materials [46, 47], e.g., poor reproducibility and degradated performance of carbon paste [48], or expensive and complicated fabrication of noble-metals (e.g., gold and platinum) [49]. Owing their hydrophilic and electrochemical properties, high surface to volume ratio, metal carbide (MXene) as a new family of two-dimensional materials has attracted significant interests and been demonstrated as an efficient electrochemical transducer [50, 51]. In virtue of good selectivity and sensitivity, enzymatic glucose sensors based on glucose oxidase (GOx) allow the catalytic oxidation of glucose to produce hydrogen peroxide, by converting the flavin adenine dinucleotide (FAD) cofactor in GOx to its reduced form (FADH_2_) [52, 53]. As the immobilization matrix for GOx enzyme is also crucial to prevent the denaturation of GOx and promote effective direct electron transfer (DET) [54, 55], in this regard, Au nanoparticles(AuNPs) was employed as the recognition element to improve the biocompatibility of the biosensor and to overcome insulating effect from the shell GOx protein[55, 56].

**Fig. 6:**
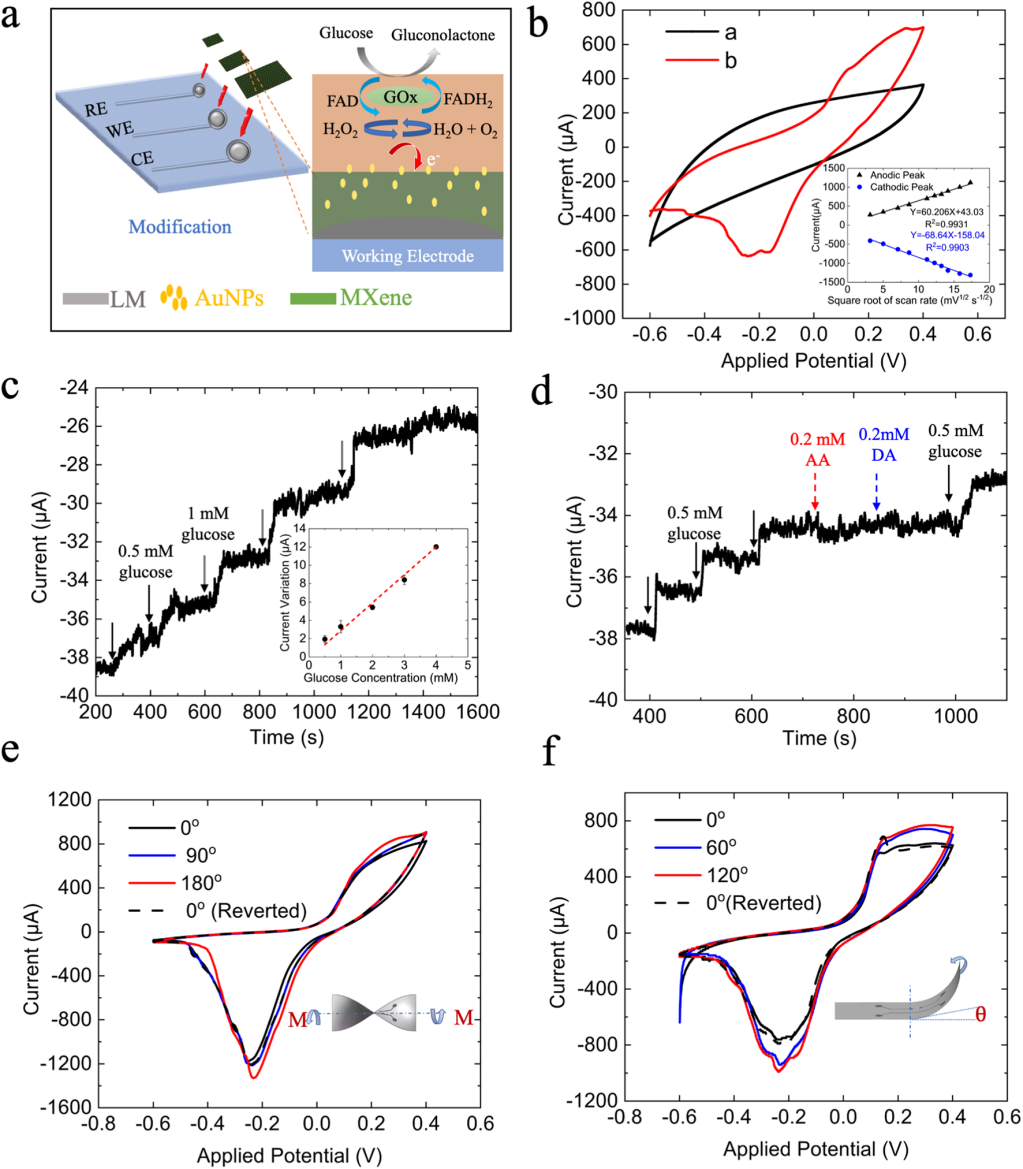
Application of LM-based flexible in biosensing **a** schematic diagram of the fabrication process of the glucose sensor. **b** Cyclic voltammograms of AuNPs/MXene/LM electrodes before (black) and after (red) immobilizing GOx in 10 mM nitrogen-saturated PBS (pH = 7.4) at scan rate of 10 mV s^−1^. Inset is the calibration curves of corresponding redox current (μA) versus square root of scan rates (mV^1/2^ s^−1/2^). **c** Typical amperometric responses and its calibration curves (inset, n = 3) of GOx/AuNPs/MXene/LM biosensor with successive additions of glucose from 0 to 4 mM in a continuously stirred (300 rpm) 10 mM PBS, applied potential: − 0.2 V, sampling interval: 0.1 s. **d** Selectivity of the glucose sensor with sequential additions of 0.5 mM glucose, 0.2 mM of ascorbic acid (AA) and dopamine (DA). **e** and **f** Cyclic voltammograms of AuNPs/MXene/LM electrodes before and after twisting (**e**) and bending (**f**) at different angles. Dash lines recorded when the electrodes restored to the unstressed state; scan rate: 100 mV s^−1^

As shown in Fig. 6a, an enzymatic biosensor, consisting of MXene solid patterns connected to the embedded LM circuits in PDMS channels, was constructed with a three-electrode configuration on which the working area was modified by GOx/AuNPs/MXene. Figure 6b illustrates the cyclic voltammetry responses before (black line) and after (red) immobilizing the GOx on the AuNPs/MXene/LM electrodes, where a pair of anodic and cathodic peaks appeared at + 0.1 V and − 0.16 V, respectively, because of immobilized redox FAD cofactor in GOx [57]. As shown in Fig. 6b(inset) and Additional file 1: Fig. S5, linear corrections between the current values and the square root of scanning rates indicated that coenzyme molecule FAD in GOx undergoes a highly reversible and diffusion-controlled process to form FADH_2_ on the surface of the LM-based biosensor. Upon successive additions of glucose in 10 mM PBS (pH = 7.4) at an applied potential of − 0.2 V, the amperometric responses (Fig. 6c) followed a good linearity (R^2^ = 0.985) in the range of 0.5–4.0 mM of glucose, with a sensitivity of 2.86 μA/mM and a detection limit of 0.21 mM (signal-to-noise ratio = 3). Benefited from the low applied potential and the enzyme catalyzed reaction, negligible current variations compared to that of glucose (shown as Fig. 6d) were obtained when adding dopamine and ascorbic acid, indicating its good selectivity and reliability for glucose analysis in complex samples towards common interferences.

Furthermore, the stability in electrochemical properties and redox behavior of GOx/AuNPs/MXene/LM electrodes under the implications of mechanical stresses, e.g., twisting and bending, plays a pivotal role in designing flexible biosensors [58–61]. Thus, we examined the influences of mechanical deformations on electrochemical behaviours of the LM-biosensors. As illustrated in Fig. 6e and f, slight increases in the redox-peak current with consistent peak separations were observed when bending or twisting the glucose sensor at different angles, which however will not impede the sensing performances [58]. After restored to the initial unstressed state, these variations in CVs and its redox behaviour became negligible, suggesting the robustness and effectiveness of the flexible LM circuits for constructions of wearable biosensors. Notably, the proposed fabrication scheme in this work holds great potential for determinising other molecules that of interests (e.g., proteins, nucleic acids, ions) in the biological, clinical and patient monitoring industries, by varying the sensing elements to different nanomaterials and bioreceptors.

## Conclusions

We herein developed soft microfluidic sensors using LM patterns as templets via soft lithography. This method can avoid the photolithographic process for channel fabrication and LM injection into the microfluidic channel, which significantly simplify the fabrication process. Through soft-lithographic technique, designed LM patterns can be easily duplicated and attached by different curable polymers (e.g., Eco-flex, PDMS, and PVA-H). The ellipse-like cross-section of LM circuits formed in the soft-lithographic process can significantly improve the storage ability and adhesion ability of LMs onto the substrates. Also, we demonstrated the excellent electrical performance under large-scale deformations including twisting, bending, pressing, and stretching and the recyclability over the conventional direct-transferring technique. Moreover, two proof-of-concept applications were proposed and implemented. The first one is a thermochromic vision sensor based on the good thermal conductivity of LMs, which can express three-color states corresponding to different forms of deformations. Second, a self-powering soft sensor was produced. Via the contact-electrification effect, the LM-based microfluidic sensor could detect variable motions (including vibration, beat, friction, and stretching) without any extra power supplies. Then, a concept of LM-based biosensor had been produced to detecting one biomarker- glucose. After a simple extra surface modification process upon the LM circuits fabricated by our method, the robustness and effectiveness of the flexible LM circuits for constructions of wearable biosensors are demonstrated through a series of tests. Therefore, the proposed method brings a new LM patterning technique for LM-based microfluidic electronics with great potentials in biosensing, soft robotics, soft batteries, and biomarkers detection.

## Experiment section

Materials: The EGaIn (Ga, 75.5%, and In, 24.5% by weight), NaOH, oxalic acid was purchased from Shanghai Macklin Biochemical Co., Ltd., Shanghai, China. The PDMS (Sylgard 184, Dow-Corning, USA), silicone of Ecoflex 00–30 (Smooth-On), PVA (205, 87.0–89.0% hydrolyzed) was used to prepare soft substrates. Commercial thermochromic pigments were purchased from Shenzhen Qiansebian Pigments Co., Ltd.

Ti_3_C_2_ (MXene, 10 mg/mL) and Gold nanoparticles (AuNPs, 0.1 mg/mL, diameter: ~ 20 nm) were purchased from Tianjin Lotov Industry Co.Ltd., Tianjin, China. Glucose oxidase (GOx, aspergillus niger, > 143 U/mg), Dopamine hydrochloride (DA), Ascorbic acid (AA), d-(+)-glucose powder, phosphate buffered saline (PBS, 0.1 M, pH = 7.4) and Nafion™ (5 wt% in mixture of lower aliphatic alcohols and water) were purchased from SigmaAldrich(Australia). All of the solutions were prepared with Milli-Q water.

Fabrication of soft substrates: (a)*.*The PDMS: base and curing agent were mixed by mechanical stirring method and then was degassed in a vacuum desiccator (ADP310C, Yamato Scientific Co., Ltd., Japan), finally cured at room temperature; (b) The Ecoflex: two parts of Ecoflex00–30 A and Ecoflex00–30 B were mixed 1:1 by weight and cured at room temperature for half an hour; and (c) The PVA-H: Glycerol, PVA, and DI water were mixed at a mass ratio of 1:6:30, and the mixture was stirred at 80 °C for 2 h, followed by degassing in a vacuum desiccator (ADP310C, Yamato Scientific Co., Ltd., Japan), followed by cooling and curing at room temperature for 24 h.

Preparation of thermochromic sensor: The thermochromic elastomer was obtained by mechanical mixing of red thermochromic pigments, green thermochromic pigments, Ecoflex00–30 A and Ecoflex00–30 B with a ratio of 1:1:50:50 by weight. In this section, a LM line (length = 60 mm, linewidth = 1 mm) was duplicated and packaged into the thermochromic elastomer.

Preparation of glucose biosensor: To obtained a three-electrode configurations, the PDMS substrate with predesign array patterns (Circular with diameter 4 mm for working and counter electrodes, and 2 mm for the reference electrode) via soft lithography were inked with 10 mg/mL Ti_3_C_2_ MXene solution, followed by overnight air-drying to remove all remaining moisture content. 20 μL of nanocomposition solution containing 0.5 mg/mL MXene, 0.01 mg/mL AuNPs and 0.5 wt% Nafion™ was ultrasonicated for 1 h at 4 °C before film cast onto the area of working electrode. Then, 40 U of GOx solution was dropcasted on the surface of AuNPs/MXene and allowed dried overnight at 4 °C. After gently washed the pretreated electrode surface to remove loosely immobilized enzyme, 5 μL of 0.2 wt% Nafion was dropped and dried on the surface of GOx/AuNPs/MXene. Afterward, the LM-based biosensor was stored in 10 mM PBS in refrigerator when not in use.

Characterization and measurements: A optical industrial microscope was employed to observe the surface structures of the LM line. The resistance changes of the LM circuits and the voltage signals of sensors were measured by a digital multimeter (DMM 6500, Keysight, USA). Electrochemical measurements were carried out on a PGSTAT128 potentiostat (Metrohm, Australia). Cyclic voltammetry and chronoamperometric characterizations were conducted in 30 mL of continuously stirred (300 rpm) PBS buffer (10 mM, pH = 7.4) at room temperature (~ 18 °C).

Softwares: The patterns and circuits on the laser engraved adhesive sheet was designed and drafted by AutoCAD (Autodesk Inc., San Rafael, CA, USA). These measured resistance changes were processed and calculated by software OriginPro2019b (origin lab corporation. MA 01060, the US). The data acquisition software supporting DMM 6500 is KICKSTARTFL_DMM authorized by Keysight. A mobile phone application FLIR ONE (support for IOS system, MacroPinch Ltd., USA) was used to measure the real-time temperature, and FLIR Tools (support for IOS system, MacroPinch Ltd., USA) was used for data processing to obtain the average temperature values.

## Supplementary Information

**Additional file 1: Figure S1.** The mechanism of the spreading of LM on the Cu surface. **Figure S2.** Contact angles on adhesive sheet and masked copper tape. **Figure S3.** The effect of height of channels on LM patterning process. **Figure S4.** A repeatability test of self-powering sensor. **Figure S5.** CVs of GOx/AuNPs/MXene modified liquid metal electrode at different scan rates: 10 to 300 mV/s. **S1.** Discussion about the feature size limitation. **S2.** The applied pressure in pressing and tensile tests. **S3.** The comparison of PDMS and Ecoflex in fabricating performance.

## Data Availability

All data generated or analyzed during this study are included in this manuscript and its additional file.
