# Supplementary material for: Construction of liquid metal-based soft microfluidic sensors via soft lithography
Source: J Nanobiotechnology. 2022 May 28;20:246. doi: 10.1186/s12951-022-01471-0 (PMC9148490; doi:10.1186/s12951-022-01471-0)
Supplement: Supplementary file 1 — Additional file 1: Figure S1. The mechanism of the spreading of LM on the Cu surface. Figure S2. Contact angles on adhesive sheet and masked copper tape. Figure S3. The effect of height of channels on LM patterning process. Figure S4. A repeatability test of self-powering sensor. Figure S5. CVs of GOx/AuNPs/MXene modified liquid metal electrode at different scan rates: 10 to 300 mV/s. S1. Discussion about the feature size limitation. S2. The applied pressure in pressing and tensile tests. S3. The comparison of PDMS and Ecoflex in fabricating performance. [file 12951_2022_1471_MOESM1_ESM.docx]

**Additional file 1**

**Construction of liquid metal-based soft microfluidic sensors via soft lithography**

Yang Zhang ^1,2^, Haowei Duan^2^，Guoqiang Li ^3^, Maoyu Peng ^1^, Xing Ma ^3^, Ming Li ^2^*, Sheng Yan ^1^*

*1. Institute for Advanced Study Shenzhen University, Shenzhen 518060, China*

*2. School of Engineering, Macquarie University, Sydney, NSW 2109, Australia*

*3. Shenzhen Key Laboratory of Flexible Printed Electronics Technology, Harbin Institute of Technology of China (Shenzhen), Shenzhen 518055, China*

***Corresponding authors.**

**Email:** [**shengyan@szu.edu.cn**](mailto:shengyan@szu.edu.cn)**;**

**ming.li@mq.edu.au**

**Table of contents**

**Figure S1** The mechanism of the spreading of LM on the Cu surface. S-3

**Figure S2** Contact angles on the adhesive sheet S-4

**Figure S3** The effect of height of channels on LM patterning process S-5

**Figure S4** A repeatability test of self-powering sensor S-6

**Figure S5** CVs of GOx/AuNPs/MXene modified liquid metal electrode at different scan rates S-7

**S1** Discussion about the feature size limitation S-8

**S2** The applied pressure in pressing and tensile tests S-10

**
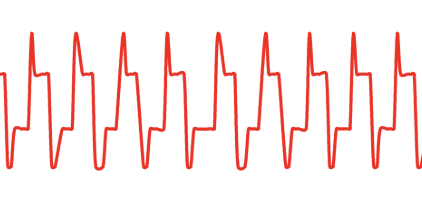

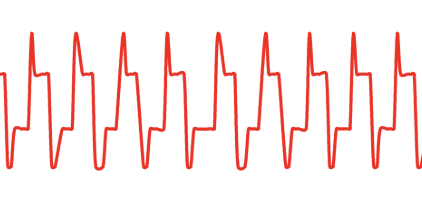
S3** The comparison of PDMS and Ecoflex in fabricating performance  **S-11**

**Figure S1**: The mechanism of the spreading of LM on the Cu surface. Note CuGa_2_@Cu represents the interface of CuGa_2_ and Cu copper.

When we applied a 5V DC voltage to the system, the electric potential difference nearby the contact line generates a localized directional flow. Because LM on CuGa_2_@Cu surface shows better wetting performance than the one on Cu surface ^33^, the total force from the electric field exhibits a driving force, which can overcome the maximum of static friction and viscous forces, leading to the spreading of LM. In addition, the NaOH solution can help to remove the oxide layer, thus accelerating the process of Ga and Cu atoms mutual penetration and diffusion, which can also be beneficial to the movement of LM on Cu.

**Figure S2**: Contact angles on the adhesive sheet (a) and masked copper tape (b). The spatial structure of the interface between LM and substrates leads to a smaller apparent contact angle θ_app_.

*
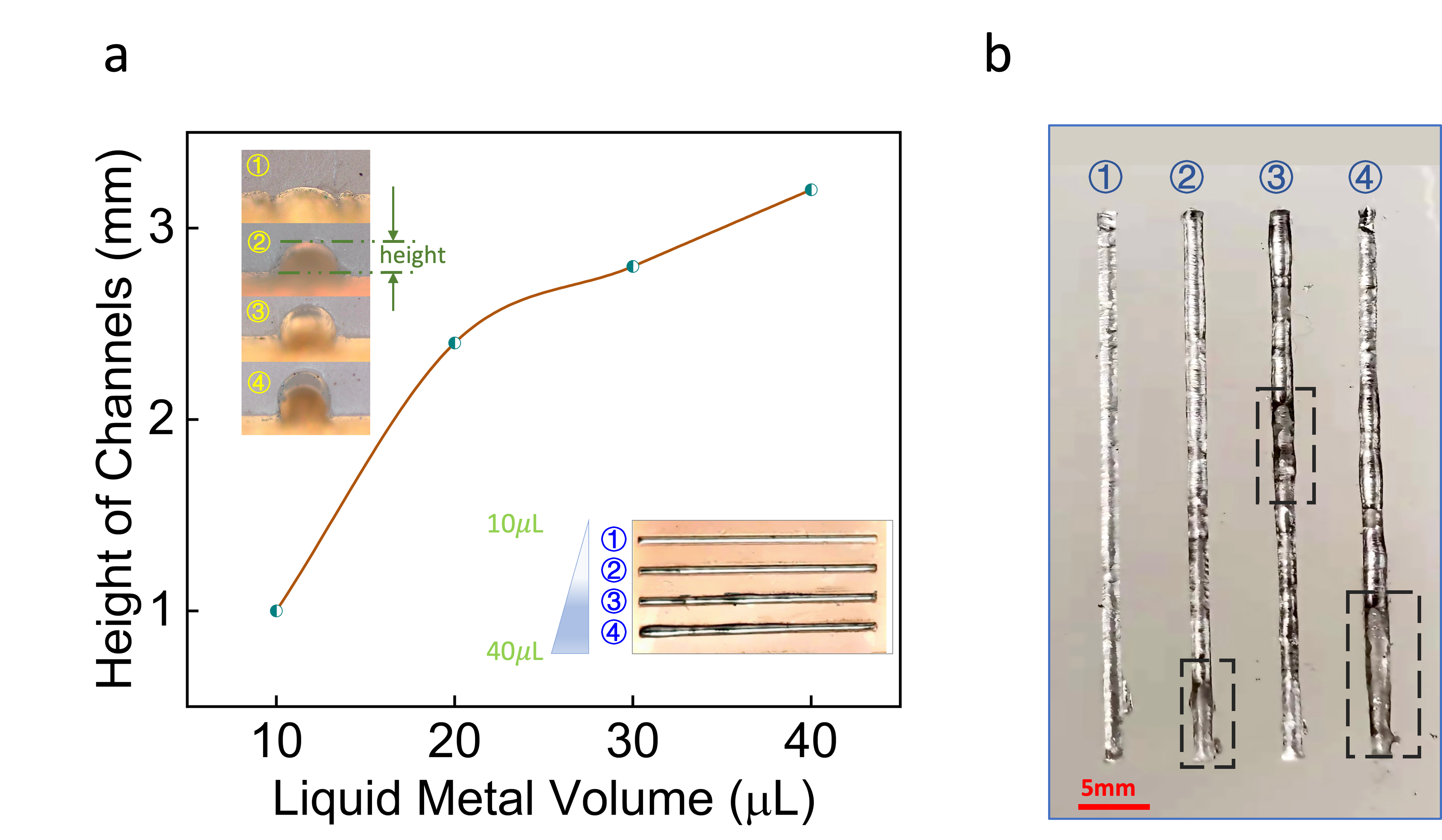
*

**Figure S3.** The effect of height of channels on LM patterning process. (a)The relationship between the LM volume and height of channels (the height was measured by an optical tensiometer after removal of LM). (b) Optical image of the LM lines on Ecoflex substrate. The black dotted boxes represent the mentioned phenomenon of unevenness on the duplicated LM lines.


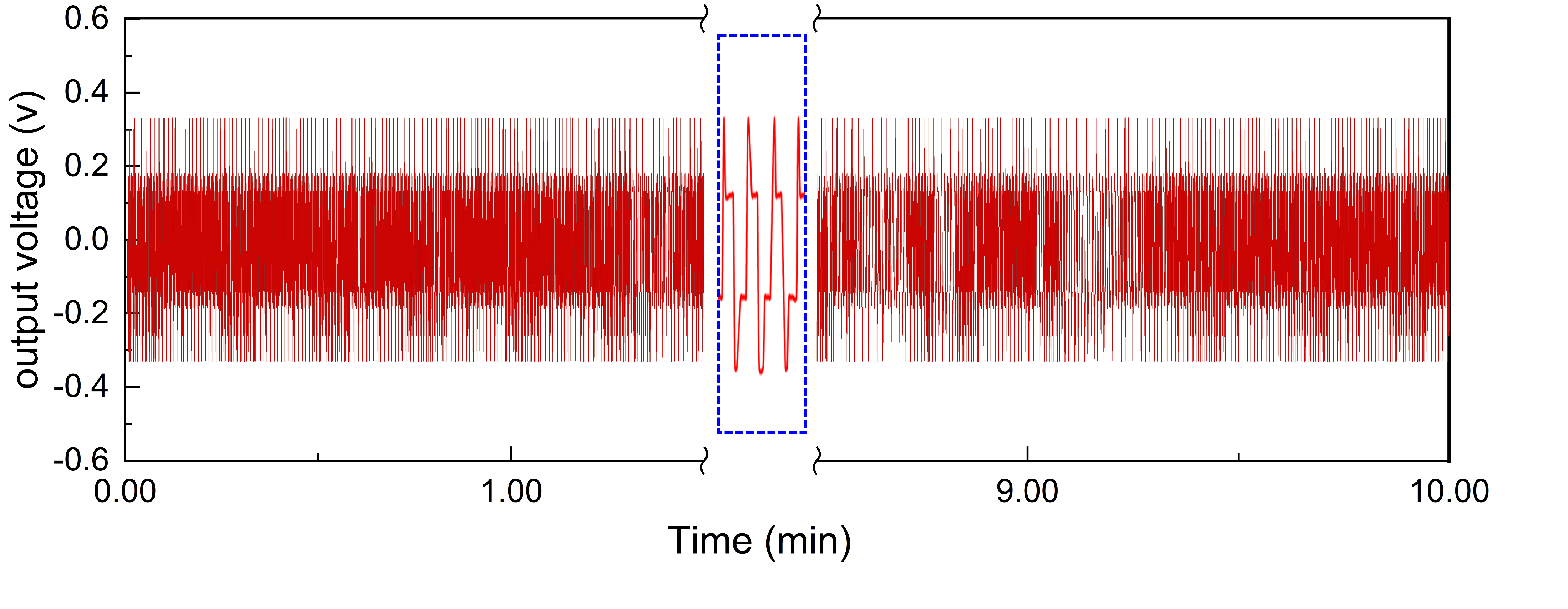


**Figure S4** The continuous monitoring of heartbeats for 10 minutes showing the repeatability of the self-powering sensor.


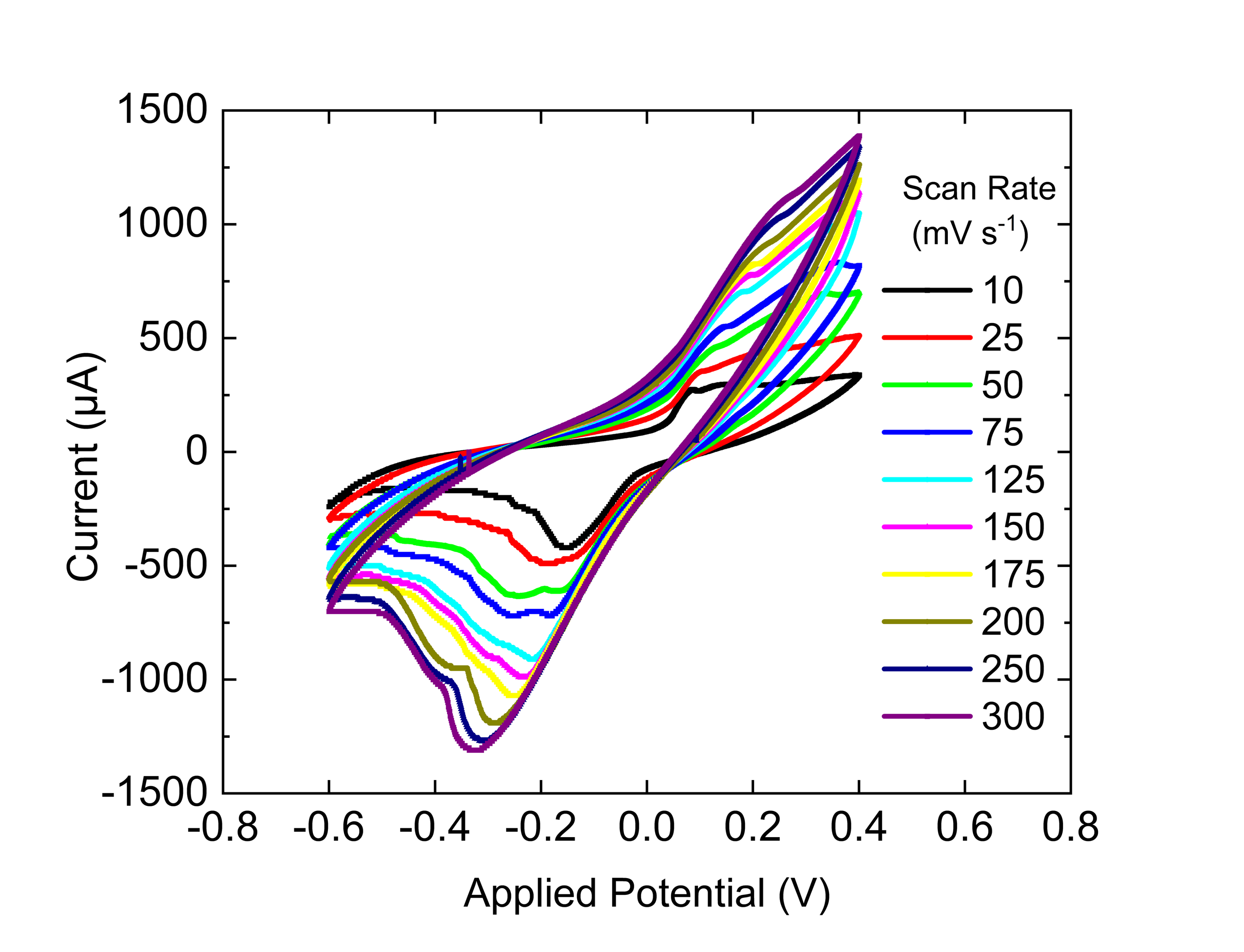


**Figure S5** CVs of GOx/AuNPs/MXene modified liquid metal electrode at different scan rates: 10 to 300 mV/s

**S1. Discussion about the feature size limitation**

**Figure S6** Schematics of the Laplace additional pressure Δ*P* (a) and the feature size of the fabricated LM line (b).

The direction of the force on the surface of the LM line points to the center of curvature (o) as shown in **Figure S6a**. If there was a change in the radius of curvature (*r*), the surface energy Δ*E* of LM circuits can be calculated using eq. (1).

$\Delta E=\sigma\cdot dA=\sigma\cdot2\pi r\cdot L\cdot dr$ (1)

where *L* is the length of LM circuits as shown in **Figure S5b**, *A* is the surface area of LM circuits. On the other hand, because of the additional pressure Δ*P* acting on the surface of LM, the work of Δ*P* can be expressed as the following eq. (2).

$\Delta W=\Delta P\cdot A\cdot dr=\Delta P\cdot4\pi r^{2}\cdot dr$ (2)

According to the law of conservation of energy, the change of surface energy is equal to the work of Δ*P*. Then, the relationship between Δ*P* and *r* can be obtained as eq. (3):

$\Delta P=\frac{2\sigma}{r} \Rightarrow\Delta P\propto\frac{1}{r} \Rightarrow\Delta P\propto\frac{1}{d}$ (3)

However, when the value of *r* is less than a critical value, the added LM cannot touch with the Cu base, which can lead to the failure of LM spreading as shown in **Figure S7**.

**Figure S7** A schematic of the gap between LM and Cu base when the linewidth *d* of the mask is less than the critical value.

The critical value using LM without any meditation was found in our experiments, which is approximate 0.3 mm. Moreover, the critical value is also related to the surface tension.

**
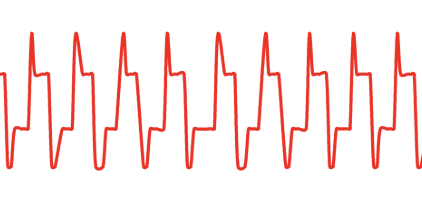

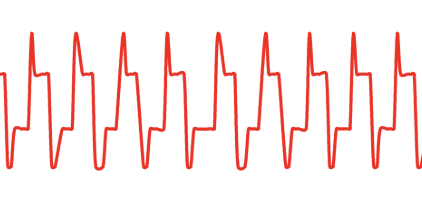
S2. The applied pressure in pressing and tensile tests**

In the case of our tests, intensity of pressure *P*, defined as the external normal force per unit area, would be selected as measured value. Then, we calculated the intensity of press *P_1_* in the pressing test in the following eq. (1). The contact surface during the pressing test was a circle with a diameter of 1 mm.

$P_{1}=\frac{F_{1}}{S_{1}}=\frac{F_{1}}{\pi{\times\frac{d}{2}}^{2}}=\frac{5\sim25 N}{\pi{\times\frac{(0.001 m)}{2}}^{2}}\approx0.64\sim3.18\times{10}^{7}Pa.$ （1）

in which, *F_1_* is the pressing force, *S_1_* is the forced area during pressing, and *d* is the diameter of forced area.

Similarly, the area of forced surface in the stretch test was the cross-section area of the sensor, which is a rectangular region (30mm × 2mm). The intensity of press *P_2_* can be calculated as eq. (2).

$P_{2}=\frac{F_{2}}{S_{2}}=\frac{F_{2}}{a\times b}=\frac{2\sim50 N}{0.03m\times0.002m}\approx0.67\sim8.3\times{10}^{5}Pa.$ (2)

in which, *F_2_* is the value of tensile force with the range from 2 *N* to 50*N*, *S_2_* is the tensile forced area, *a* and *b* is the length and width of the rectangular cross-section, respectively.

As mentioned in the manuscript, the RRV in the pressing test rase by ≈ 5000% while that increased 170% in the tensile test. The RRV difference between stretching and pressing can attribute to the difference between *P_1_* and *P_2_*.

**
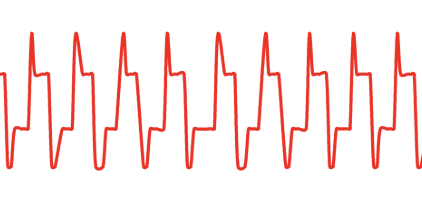

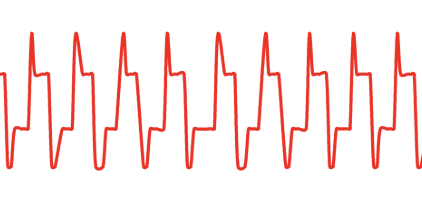
S3. The comparison of PDMS and Ecoflex in fabricating performance**

We firstly listed the difference parameters related our applications in this work in **Table S1**. To sum up, the Ecoflex has a lower Young’s module than PDMS, which means the Ecoflex materials would generate larger deformation response than PDMS with the same external excitation. In other words, Ecoflex encapsulated LM-based flexible sensors are much more sensitive in detecting deformation signal. The working mechanism of thermochromic sensor and self-powered soft sensor in our research is that transferring deformation signal into thermal and electricity signal as output, respectively. That’s also the reason why Ecoflex was employed when we did demo experiments in the two cases. In addition, the thermal conductivity of Ecoflex is greater the PDMS, which lead to better heat transfer efficiency and resolution improvement while be utilized to thermochromic sensing. Moreover, the Ecoflex encapsulated sensor has a much larger working range because of its 4 times higher elongation as failure than PDMS. In contrast, the LM circuits in glucose detecting biosensor would try to keep stable when suffering external pressure and different forms of deformation, thus we can detect a relative correct electro-chemical signal. Therefore, the PDMS was chosen to encapsulate the biosensor, which can be not only as a flexible substrate to achieve wearable function, but can reduce the leakage of LM due to the higher Young’s modules.

**Table 1** The comparison of material properties between PDMS and Ecoflex

|  | PDMS | Ecoflex |
| --- | --- | --- |
| Young’s module (Kpa) | 400~750 | ~125 |
| Elongation at failure | ~190% | ~ 900% |
| Thermal conductivity  W/(m•k) | 0.134 ~ 0.159 | 0.20 ±0.01 |
| Curing time  (room temperature) | >48 hours | ~ 4 hours |

”
